# Asymmetric Dimethyl Arginine as a Biomarker of Atherosclerosis in Rheumatoid Arthritis

**DOI:** 10.1155/2018/3897295

**Published:** 2018-01-18

**Authors:** Manuela Di Franco, Bruno Lucchino, Fabrizio Conti, Guido Valesini, Francesca Romana Spinelli

**Affiliations:** Dipartimento di Medicina Interna e Specialità Mediche-Reumatologia, Sapienza Università di Roma, Rome, Italy

## Abstract

Cardiovascular disease is the main cause of morbidity and mortality in rheumatoid arthritis (RA). Despite the advent on new drugs targeting the articular manifestations, the burden of cardiovascular disease is still an unmet need in the management of RA. The pathophysiology of accelerated atherosclerosis associated to RA is not yet fully understood, and reliable and specific markers of early cardiovascular involvement are still lacking. Asymmetric dimethylarginine is gaining attention for its implication in the pathogenesis of endothelial dysfunction and as biomarkers of subclinical atherosclerosis. Moreover, the metabolic pathway of methylarginines offers possible targets for therapeutic interventions to decrease the cardiovascular risk. The purpose of this review is to describe the main causes of increased methylarginine levels in RA, their implication in accelerated atherosclerosis, the possible role as biomarkers of cardiovascular risk, and finally the available data on current pharmacological treatment.

## 1. Introduction

Patients with rheumatoid arthritis (RA) have a significantly higher risk of cardiovascular diseases (CVD) compared to general population, comparable to patients with diabetes mellitus or non-RA subjects 10 years older [1]. In RA patients, cardiovascular events account for over 50% of the excess premature mortality [2]. Accelerated atherosclerosis plays a pivotal role in the pathogenesis of RA-related CVD: indeed, in RA patients, the atherosclerotic process starts in the early phases of the disease and it is determined by both an increased prevalence of traditional risk factors and the inflammatory nature of RA itself [3, 4]. The systemic inflammation has a major role in the pathogenesis of accelerated atherosclerosis. Proinflammatory cytokines involved in the pathogenesis of RA, such as TNF, IL-1, and IL-6, are also involved in the development and in the progression of atherosclerotic plaque. The first step in plaque development is the activation of endothelial cells and the induction of endothelial dysfunction (ED) by proinflammatory cytokines. The proatherogenic and prothrombotic endothelium is characterized by upregulation of adhesion molecules, raised vascular permeability, cytokine and chemokine expression, and reduced production of vasodilatory molecules, such as nitric oxide [5]. ED is the earliest, reversible, preclinical phase of plaque development, leading to the accumulation of lipoproteins and inflammatory cells in the subendothelial layer and to subsequent plaque formation [5]. Other than activating endothelial cells, TNF and IL-6 activate monocytes and immune cells contributing to the progression of the atherosclerotic disease, until rupture and thrombotic complication of the plaque [6]. There is a growing interest around the prevention of CVD in RA patients, although there is no clear evidence that any intervention can actually reduce that risk [7]. Early identification of ED may allow clinicians to characterize patients with subclinical atherosclerosis, establishing early risk factor modification or pharmacological intervention [5]. The imbalanced production of endothelial vasoactive mediators is a key step in the development of ED. Nitric oxide (NO) is the main endothelial-derived vasodilatory and antiproliferative molecule, inhibiting activation and vessel wall adhesion of leukocytes and platelets [8]. The impaired ability of endothelial cells to produce NO is a main driver of ED. Dysregulation of other vasoactive mediators of NO metabolism predispose to subsequent pathological abnormalities such as platelet activation, abnormal fibrinolytic activity, lipoprotein deposition, and oxidative stress: all these modifications contribute to impaired vascular integrity [5, 9]. The role of endogenous inhibitors of NO synthase (NOS) activity in the induction of ED has gained the attention of rheumatologists. Asymmetric dimethylarginine (ADMA) is an analogue of L-arginine—the precursor of NO—naturally released in biological fluids following proteolysis; it inhibits NO synthesis by competing with L-arginine at the active site of NOS [10]. ADMA emerged as novel markers of ED and cardiovascular risk in RA [11]. The aim of this review is to summarize the available data on the role of ADMA in the pathogenesis of ED in RA patients, its role as potential biomarkers of CVD risk, and the possible therapeutic interventions.

## 2. Methylarginine Metabolism

**Figure 1:**
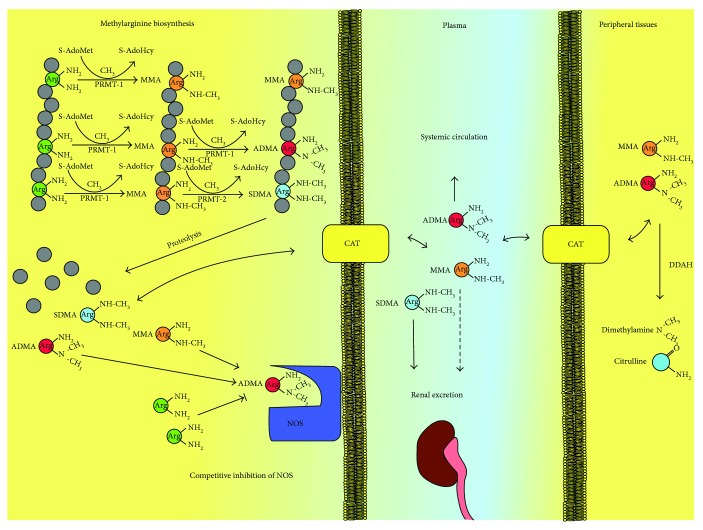
Metabolic pathways of methylarginines. S-AdoMet: S-adenosyl-l-methionine; S-AdoHcy: S-adenosylhomocysteine; PRMT: protein arginine methyltransferases; MMA: monomethyl arginine; ADMA: asymmetric dimethyl arginine; SMDA: symmetric dimethyl arginine; CAT: cationic amino acid transporter; DDAH: dimethylarginine dimethylaminohydrolase; NOS: nitric oxide synthase.

Dimethylarginines are naturally occurring endogenous products of the degradation of methylated proteins. Methylation of arginine residues is a posttranslational modification catalyzed by a family of enzymes called protein arginine methyltransferases (PRMTs) which use S-adenosylmethionine as source of methyl groups; methylation of arginine is a two-step process of monomethylation [12, 13]. The first methylation leads to the formation of monomethylarginine (MMA), while the second one can produce either symmetric dimethylarginine (SDMA) or ADMA, according to the PRMT isoform involved in the methylation reaction [14]. After their proteolysis, MMA, SDMA, and ADMA are released in the cytosol, where the asymmetric methylarginines (MMA and ADMA) inhibit NOS activity by competing with L-arginine for the active site of the enzyme [15]. Cationic amino acid transporters (CATs) are the transmembrane enzymes which carry out methylarginines and arginine from the cellular cytosol to extracellular fluids and then in the bloodstream [16]. In physiological conditions, intracellular levels of arginine are much higher than those required for NOS activity; however, intravenous supplementation of arginine can increase endothelial-dependent vasodilatation [17]. This apparently incongruous phenomenon is called “arginine paradox”: several hypotheses have been proposed to explain this effect. The activity of the enzyme arginase, which converts arginine in ornithine and urea, may reduce the availability of arginine, decreasing NOS activity. However, arginine is converted by NOS in an intermediate state, the hydroxy-L-arginine, which inhibits arginase, increasing substrate bioavailability for NOS. Another possible explanation is the competitive occupation of CATs by arginine excess for intracellular space transportation instead of other cationic amino acids [17]. CATs and NOS are located in the plasmatic membrane caveolae, ensuring a stable supply of the substrate (i.e., arginine) from the plasmatic compartment [18]. A relative abundance of plasmatic arginine may overtake NOS inhibition by raising intracellular arginine/ADMA ratio in the strict proximity of NOS [19]. However, using the same transporter, plasmatic ADMA may also gain a selective access to NOS, thus reducing NO bioavailability and explaining the association with the ED and, subsequently, with the increase in cardiovascular risk [16]. Once in the circulation, methylarginine can be eliminated through renal excretion or tissue catabolic pathways [13]. About 20% of ADMA is removed from plasma by the kidney while SDMA is mostly excreted unmodified through the urine [20]. The main pathway for asymmetric methylarginine catabolism is the hydrolytic reaction mediated by dimethylarginine dimethylaminohydrolase (DDAH) enzymes which catalyze the degradation of MMA and ADMA to citrulline and monomethylamine or dimethylamine, respectively [21]. Different tissues and cells express DDAH including heart, endothelium, kidney, lung, pancreas, liver, brain, and placenta as well as macrophages and neutrophils; however, ADMA is mostly catalyzed by the kidney and liver [22, 23]. A further catabolic pathway for both symmetric and asymmetric methylarginines is the transamination mediated by alanine-glyoxylate aminotransferase; however, the contribution of transamination to ADMA metabolism has not been fully investigated [24]. The methylarginine metabolism is depicted in Figure 1.

## 3. Physiopathology of ADMA and Endothelial Dysfunction in Rheumatoid Arthritis

### 3.1. Factors Affecting ADMA Levels in RA Patients

Different mechanisms can account for the increase in ADMA levels detected in RA patients. The inducible NOS (iNOS) is an isoform that can be induced in various cellular types under inflammatory stimuli; iNOS has a crucial role in the intracellular clearance of pathogens and in the vasodilatation of inflamed tissues [25]. However, the increased production of NO by iNOS, primed by inflammatory cytokines, leads to an S-nitrosylation of reactive cysteine in DDAH, inhibiting ADMA catabolism, thus increasing its levels and lastly inhibiting all three isoforms of NOS [26]. *In vitro* studies on endothelial cells demonstrated that TNF, a cytokine playing a key role in RA pathogenesis, exerts an inhibitory effect on DDAH leading to the impairment in ADMA degradation [27]. In RA patients, free radicals and nitrotyrosine produced by rheumatoid synovia as well as by the reduced expression of DDAH enzyme in the hypoxic environment of inflamed synovia may further contribute to DDAH inhibition and rise in plasmatic ADMA levels [28–30]. Another explanation for the high ADMA levels is an increase in its production by PRMT activity: Böger et al. described an enhanced production of ADMA in endothelial cells exposed to native and oxidized LDL (oxLDL), partially due to enhanced PRMT gene expression [31]. oxLDL levels are higher in RA patients than in healthy subjects because of the oxidative stress coexisting with the inflammatory state [32, 33]. Moreover, other posttranslational modifications of LDL may also account for NO uncoupling [34]. In the rheumatoid synovia, endothelial cells undergo a phenotypic change characterized by an increase in activation, angiogenesis, and apoptosis [35]. The increased turnover of endothelial cells as well as the increased number of proliferating cells associated with angiogenetic microenvironment of the inflamed joint may be a source of methylarginines. ADMA production is enhanced in apoptotic and senescent endothelial cells, as a result of methylated protein turnover [36].

Patients with RA have a high basal level of insulin and a tendency toward insulin resistance which is associated with the inflammatory status and seems to be reverted by TNF inhibitors [37, 38]. Proinflammatory cytokines such as TNF and IL-6 prevent muscular glucose uptake and induce lipolysis in adipocytes, leading to an impaired plasmatic glucose regulation; moreover, free fatty acids released by stimulated adipocytes determine a positive feedback loop both by inducing an insulin-resistant phenotype of skeletal muscle and liver and by stimulating TNF and IL-6 production by macrophages [39, 40]. In diabetic patients, both increased and decreased levels of ADMA were reported [41, 42]. Chronic hyperglycemia increases ADMA levels by inhibiting DDAH activity [43]. On the contrary, insulin upregulates CAT expression in various cell types [44]. In healthy subjects and in type 1 diabetic patients, acute hyperinsulinemia reduces ADMA levels, probably increasing the cellular uptake related to CAT regulation [45, 46]. Raising the production of ADMA (via DDAH inhibition) and increasing cellular uptake (via CAT upregulation), insulin resistance may contribute to ADMA-mediated NOS inhibition [16].

Homocysteine (Hcy) is a sulfhydryl-containing amino acid mainly produced from the essential amino acid methionine. Several factors affect Hcy levels, including age, sex, lifestyle factors (coffee consumption, smoking habit, physical activity, and alcohol), genotype of the enzymes involved in Hcy catabolism, drugs and diseases interfering with its metabolism, and most importantly group B vitamins (folic acid, pyridoxine, and cobalamin) [47]. Hyperhomocysteinemia (HHcy) is a well-known risk factor for CVD in general population and in patients with RA [48, 49]. Some authors suggested a link between HHcy and increased ADMA levels; indeed, Hcy inhibits DDAH activity and the endoplasmic reticulum stress response in the dysfunctional endothelium seems to increase proteolysis, and thus ADMA levels [50, 51]. In RA patients, several factors contribute to the increase in Hcy serum levels. Chronic inflammation enhances immune cell turnover increasing the folate requirement, and the use of methotrexate contributes to folate deficiency by inhibiting the enzyme dihydrofolate reductase [52, 53]. The reduced bioavailability of the methylenetetrahydrofolate, the key substrate of methylenetetrahydrofolate reductase, limits the conversion of Hcy to methionine, causing HHcy [53]. The link between NO metabolism and HHcy is not completely clear since Hcy-lowering agents seem not to significantly affect ADMA levels [54].

### 3.2. Linking ADMA to Endothelial Dysfunction in RA

Normal endothelium is responsible for many physiological functions needed to maintain vascular integrity, such as regulation of vascular tone and anticoagulating and anti-inflammatory functions [55]. NO is a key mediator of many functions of a healthy and functional endothelium, and consequently, the impaired ability to produce NO is a main feature of ED [56]. A dysfunctional endothelium is characterized by cytokine and chemokine production, adhesion molecule expression, platelet activation, abnormal fibrinolytic activity, lipoprotein deposition, and immune cell migration in the subendothelial layer leading to the early and subclinical phases of the atherosclerosis and driving all the steps of CVD until acute complications [5, 8, 9, 55].

Methylarginines affect endothelial function in different ways. Asymmetric methylarginines inhibit the three isoforms of NOS, reducing the NO production [15]. Furthermore, ADMA and MMA can compete with arginine for transmembrane transport through CAT, reducing the availability of the substrate for NO synthesis [57, 58]. Besides the interference with arginine-dependent NO production, ADMA determine “NOS uncoupling,” a shift in NOS enzymatic activity from reductase to oxidase [59]. In the absence of its substrate, NOS transfers electrons to molecular oxygen, instead of arginine, leading to the formation of superoxide, instead of NO [59]. Superoxide is a free radical which rapidly combines with NO producing peroxynitrite, a highly reacting intermediate and powerful source of oxidative stress that entails DNA and protein oxidation and at high concentration, cytotoxicity [60]. Therefore, superoxide and peroxynitrite produced by ADMA-related NOS uncoupling contribute to oxidative stress and endothelial cell dysfunction [61].

**Figure 2:**
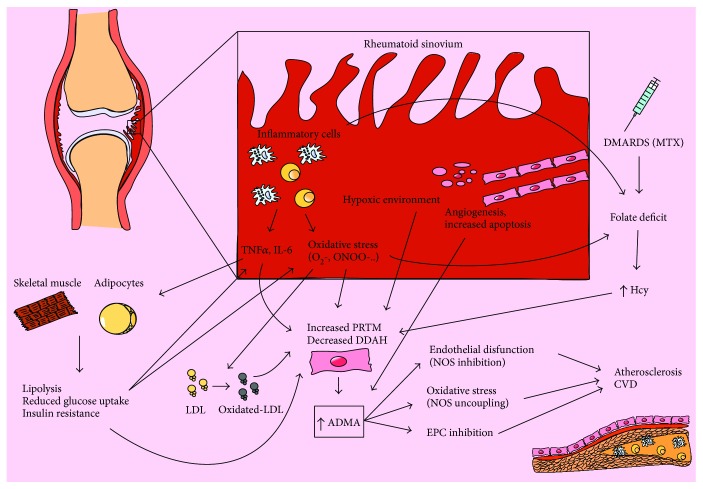
Mechanisms of ADMA-induced endothelial dysfunction in rheumatoid arthritis. The inflammatory microenvironment of the inflamed synovia produces cytokines and reactive oxygen species which directly stimulate PRTM and inhibits DDAH in endothelial cells, increasing ADMA production. Cytokines influence metabolically active tissues like skeletal muscle and adipocytes, inducing insulin resistance. This can generate a positive feedback loop, with an increased release of cytokines from macrophages and increased ADMA production in endothelial cells as well. The increased levels of oxidated lipoproteins, as a consequence of the oxidative stress linked to synovitis, furthermore contribute to ADMA synthesis, as well as the increased apoptosis of endothelial cells in inflamed synovium. At last, folate deficit, related to the increased cellular turnover, to the oxidation of folate from reactive oxygen species and to the methotrexate treatment, induces an increased generation of homocysteine, which contributes to ADMA increase. ADMA increase can induce endothelial dysfunction, oxidative stress, and EPCs inhibition, conducing atherosclerosis development and cardiovascular complications. PRMT: protein arginine methyltransferases; DDAH: dimethylarginine dimethylaminohydrolase; NOS: nitric oxide synthase; ADMA: asymmetric dimethyl arginine; EPCs: endothelial progenitor cells; MTX: methotrexate; Hcy: homocysteine; CVD: cardiovascular disease.

Endothelial progenitor cells (EPCs) are bone marrow derived, circulating endothelial precursors able to differentiate in situ in functional endothelium, contributing to endothelial injury recovery and limiting atherosclerotic plaque formation; in the light of their repairing effect, EPCs are biomarkers of endothelial health [62]. A reduced number of circulating EPCs has been described in a number of conditions associated with an increased cardiovascular risk, including RA [63, 64]. In patients with RA, different authors observed an inverse correlation between ADMA levels and the number of circulating EPCs which can be reversed by TNF inhibitors [64–67]. Since NO is a key regulator of EPC migration and differentiation, lowering endogenous production of NO by the endothelium, ADMA can markedly reduce the mobilization and function of EPCs, impairing the protective effect [67, 68].  Figure 2 summarizes the physiopathology of ADMA in ED development in patients with RA.

## 4. ADMA as Biomarker of Cardiovascular Risk in Rheumatoid Arthritis

In the last years, the potential role of ADMA as a biomarker of cardiovascular risk has been investigated in several conditions. Recently, a meta-analysis of about 20,000 nonoverlapping participants enrolled in 22 cohort studies and long-term follow-up demonstrated an association between circulating levels of ADMA and cardiovascular outcomes, including coronary heart disease and stroke [69]. ADMA was also correlated with noninvasive markers of subclinical atherosclerosis such as flow-mediated dilation (FMD) and intima-media thickness (IMT). Brachial artery FMD is a noninvasive method to evaluate NO-mediated flow response to subischemic stimuli. FMD is a useful marker of CVD risk since it correlates with more invasive measurement of ED, with cardiovascular risk, and with coronary artery vasodilatory function [70]. In healthy subjects, elevated ADMA levels are associated with a reduced FMD, suggesting that ADMA may represent a biomarker of ED [71, 72]. In RA patients, the decrease of the endothelium-dependent macrovascular function starts to be evident within the first year of the disease; some authors detected an association with disease activity, not confirmed by others, and with serology [73, 74]. Some reports suggested an inverse correlation between ADMA levels and FMD, not confirmed by other studies [66, 75–77] (Table 1).

Ultrasonographic evaluation of carotid IMT is a reliable marker of cardiovascular outcome correlating with traditional risk factors and with the incidence of clinical cardiovascular events [78, 79]. A meta-analysis of the literature published in 2015 reported an increased carotid IMT with a higher prevalence of carotid plaque in RA patients compared to control subjects [80]. A meta-analysis of over 6,000 patients showed a positive relation between carotid IMT and ADMA, suggesting a role for the latter as a serological biomarker of cardiovascular risk [81]. As for RA, literature data seems not to confirm the association between carotid IMT and ADMA levels [77, 82–84] (Table 1). A single recent study, investigating biomarkers of micro- and macrovascular function in 197 RA patients, demonstrated a significant correlation between ADMA levels and noninvasive markers of endothelial dysfunction, in those patients showing a high disease activity: the authors showed a positive correlation between ADMA levels and cIMT and between arterial stiffness and ADMA/SDMA ratio, especially in patients with high inflammatory markers [85].

The studies investigating a possible association between markers of disease activity and ADMA led to conflicting results. A few studies on RA patients demonstrated a positive correlation between ADMA levels and C-reactive protein and disease activity score (DAS28) values, suggesting a link between a high inflammatory state, ADMA levels, and CVD in active RA; however, other studies failed to replicate these results [77, 82, 86–89]. Similarly, some reports described an association between anticitrullinated peptide antibodies (ACPA) titer and ADMA levels, especially in patients with early disease [87, 90, 91]. In RA patients, ADMA showed a positive correlation with Hcy levels and it is associated with insulin resistance (the homeostasis model assessment (HOMA) being a strong predictor of ADMA serum levels) [92, 93]. Finally, ADMA serum levels correlate with other markers of endothelial health status, such as EPCs: the reduction in circulating EPCs, correlating with high plasmatic level of ADMA and high DAS28 values, was restored by a short course of TNF inhibitors [65]. In summary, these observations strongly suggest a possible role of ADMA as a reliable biomarker of early atherosclerosis in RA patients, especially in the context of an active disease.

ADMA levels start increasing in the early phase of disease, and the introduction of disease-modifying antirheumatic drug (DMARD) treatment seems to decrease the levels compared to those observed in the control group [77]. In a study on 20 early, untreated RA patients, our group demonstrated that therapeutic intervention with conventional synthetic DMARDs or TNF inhibitors significantly reduced ADMA serum levels [77]. Even in long-standing RA patients, the treatment with TNF inhibitors seems to reduce ADMA levels: this effect was shown in a study on 33 RA patients starting etanercept or adalimumab but was not confirmed by other authors [66, 83, 84, 91].  Table 1 summarizes the main findings of the studies investigating ADMA serum levels in the context of RA [65, 66, 75–77, 82–98].

The heterogeneity of methods used to assess subclinical atherosclerosis and the different contributions of traditional and disease-related risk factors in a complex disease such as RA may account for the lack of concordance of the results and limit the usefulness of ADMA as a marker for atherosclerotic risk stratification. In this regard, a cutoff level of ADMA defining a dysfunctional endothelium could be helpful.

## 5. Possible Therapeutic Intervention

Since methylarginines play a key role in the physiopathology of ED and ADMA levels have been strictly associated to cardiovascular risk, several pharmacological interventions have been investigated on the possible effect on ADMA levels and cardiovascular outcomes. However, taking into account the wide spectrum of indications of the drugs investigated and of the inter-study result variability, the actual relation between ADMA level reduction and cardiovascular benefits is still inconclusive [13]. Effect of statins on methylarginine metabolism has been investigated in different conditions such as diabetes, stroke, and hypercholesterolemia, demonstrated to effectively reduce plasmatic ADMA levels in recent controlled trials [99–101]. *In vitro*, statins increase the expression of DDAH genes and the bioavailability of tetrahydrobiopterin (BH4), which is a critical eNOS cofactor inhibiting NOS uncoupling phenomenon [102]. A recent double-blind randomized study demonstrated that supplementation of oral tetrahydrobiopterin significantly improved the endothelial function measured by FMD in a small cohort of RA patients [103]. The authors did not investigate the effect on ADMA levels but, considering the implication of folate in methylarginine metabolism, an ADMA-lowering effect could be expected. This is also supported by the consolidated evidence of the role of folate supplementation on plasmatic Hcy lowering, in consideration of the interplay between HHcy and raised ADMA levels [47]. This suggests that larger and targeted studies, addressing the potential effect of tetrahydrobiopterin supplementation on ADMA levels in relation to ED and risk of CVD, are desirable.

In a small study on RA patients, atorvastatin effectively reduced arterial stiffness measured by pulse wave analysis, without affecting acute-phase reactants [104]. The lipid-lowering agent ezetimibe showed the ability to lower ADMA levels and to ameliorate renal function in patients with chronic kidney disease, probably by protecting DDAH enzymatic site from oxidative inactivation [105]. Besides the lipid-lowering effect, ezetimibe, as well as simvastatin, demonstrated to reduce disease activity and C-reactive protein levels and to improve the endothelial function and the arterial stiffness in patients with RA [106].

The evidence that lipid-lowering drugs couple an anti-inflammatory effect with an improvement of endothelial function, by modulation of ADMA metabolism, may suggest a role for these drugs in the management of cardiovascular risk associated to RA. The ADMA-lowering effect of several other agents have been investigated in conditions different from RA. Only few studies addressed the effects of therapeutic intervention for RA on ADMA levels. Treatment with DMARDs, especially anti-TNF agents, demonstrated a lowering effect on ADMA levels, more pronounced in high inflammatory conditions (patients with high levels of acute-phase reactants) [85]. A recent meta-analysis showed that treatment with TNF inhibitors improves endothelial function in patients with RA [107]. It is very likely that effect of TNF inhibitors on cardiovascular risk is multifactorial, acting on different steps of the atherosclerotic process. Longitudinal studies demonstrated a short-term effect of TNF inhibitors on ADMA levels, not confirmed in studies with different follow-up [77, 83, 84]. Nevertheless, in a 12 month follow-up study, TNF inhibitors improved the arginine/ADMA ratio despite not impacting on ADMA absolute levels [84]. These results imply that the modulation of ADMA metabolism could partially account to the atheroprotective effect of TNF inhibitors.

The effect of folate supplementation on plasmatic Hcy is well known and some authors hypothesized an interplay between HHcy and raised ADMA [47]. A single study on a large population of RA patients (*n* = 201) demonstrated that Hcy levels are significantly related to serum ADMA, contrasting with previous data obtained in a smaller group of patients [92, 98]. The relationship between ADMA and Hcy levels is intriguing since the latter is affected by the use of methotrexate, a milestone in the RA treatment. In a recent study, Dimitroulas et al. demonstrated a trend of the MTHFR polymorphism to influence ADMA levels, with the C667T polymorphism associated to higher ADMA levels, only at the univariate analysis [92]. Interestingly, C677T polymorphism was associated with subclinical atherosclerosis and CVD risk in a study on 612 RA patients followed up for 5 and 10 years [108]. These evidences may support the protective, antiatherogenic effect of methotrexate.

A very recent study investigated the effect of low-dose glucocorticoids on arginine metabolisms by comparing patients who were chronically treated or not with prednisolone and demonstrated higher levels of ADMA and MMA in those patients who were not taking glucocorticoids; the authors conclude that long-term glucocorticoid treatment could help in protecting endothelial health in RA patients [109].

Table 2 summarizes potential therapeutic intervention with ADMA-lowering effect.

## 6. Conclusion

CVD risk reduction is still an unmet need in the long-term management of RA patients and, despite the great improvement of RA treatment, CVD is still the main cause of death. In 2016, the European League Against Rheumatism (EULAR) updated the recommendations for the management of CVD in rheumatic disease firstly published in 2009, suggesting the need for an aggressive and targeted risk management [110]. The research agenda still includes issues about the precise effect of antirheumatic drugs with different modes of action and the additional value of novel biomarkers for CVD risk prediction on CVD risk [110]. The physiopathology of ED in chronic inflammatory diseases such as RA is still largely unknown, and biomarkers to efficiently stratify patients according to their CV risk are scant. ADMA seems to have the potential to solve part of these issues. The apparent physiopathological role of ADMA in endothelial NO deficit as well as the correlation between the circulating ADMA levels and cardiovascular outcomes suggest that ADMA could be a good candidate for further basic research. Moreover, better understanding the role of ADMA in ED could also provide potential target of pharmacological intervention to lower the cardiovascular risk in RA.

## Figures and Tables

**Table 1:** Main findings of the studies investigating ADMA in rheumatoid arthritis.

Number of RA patients (controls)	Main findings	Reference
91 (31)	No correlation between ADMA and subendocardial viability ratio	Anyfanti et al. [94]
201	No association between ADMA and genetic variants of the AGXT2 gene	Dimitroulas et al. [95]
197	Association between microvascular function, arterial stiffness, and cIMT and ADMA/SDMA levels in RA patients with high inflammatory marker	Dimitroulas et al. [85]
40 (29)	Inverse correlation between ADMA and FMD; positive correlation between ADMA and disease duration; no correlation with CRP	Sentürk et al. [88]
30 (30)	No relationship between ADMA concentration and aortic augmentation; no difference in ADMA levels between patients and controls	Erre et al. [96]
201	Difference in ADMA levels according to MTHFR; positive correlation between ADMA and Hcy and ESR	Dimitroulas et al. [92]
100	No correlation between ADMA and thCys at baseline and after omega-3 fatty acids, vitamin E, vitamin A, copper, and selenium, or placebo; correlation between ADMA and arginine	Kayacelebi et al. [98]
201	Positive correlation between ADMA and ESR and ADMA and CRP	Sandoo et al. [89]
33	Correlation between ADMA and DAS28; reduction of ADMA levels after 3 months of anti-TNF	Spinelli et al. [66]
201	No significant relationship between DDAH genetic variables and ADMA levels	Dimitroulas et al. [97]
17 (12)	Inverse correlation between ADMA levels and circulating EPC number	Spinelli et al. [65]
35 (35)	ADMA and RF have similar sensitivity and specificity in the detection of endothelial dysfunction	Spasovski and Sotirova [91]
67	HOMA, an indicator of insulin resistance, predicts elevated ADMA levels	Dimitroulas et al. [93]
48 (32)	Association between baseline PWV and ADMA but no correlation with cIMT; anti-TNF therapy increased L-arginine/ADMA ratio but not ADMA after 3 months	Angel et al. [84]
20 (20)	Significantly higher ADMA levels in RA than controls; significant reduction after 12 months of treatment	Di Franco et al. [77]
35	No change in ADMA levels after 2 weeks and 3 months of anti-TNF treatment	Sandoo et al. [75]
46 (50)	Higher ADMA levels in RA than in controls; correlation with CRP, DAS28, and 8-isoprostanes	Kwaśny-Krochin et al. [86]
60 (29)	Significantly higher ADMA levels in RA compared with controls; no correlation with demographic or disease characteristics	Sandoo et al. [83]
25	No change in ADMA levels and cIMT after treatment	Turiel et al. [82]
25 (25)	Higher ADMA levels in early RA than in controls. Significant negative correlation between ADMA levels and CFR; no correlation with IMT	Turiel et al. [90]
20	Positive correlation between ACPA and ADMA levels; no correlation with disease activity indices	Surdacki et al. [87]
36 (20)	Chronic low-dose prednisolone lower ADMA levels	Radhakutty et al. [109]

ADMA = asymmetric dimethyl arginine; AGXT2 = alanine-glyoxylate aminotransferase 2; SDMA = symmetric dimethyl arginine; cIMT = carotid intima media thickness; FMD = flow-mediated dilation; CRP = C-reactive protein; MTHFR = methylenetetrahydrofolate reductase; Hcy = homocysteine; ESR = erythrocyte sedimentation rate; thCys = total L-homocysteine; DAS28 = disease activity score 28; TNF = tumor necrosis factor; DDAH = dimethylaminohydrolase; EPCs = endothelial progenitor cells; RF = rheumatoid factor; HOMA = homeostasis model assessment; PWV = pulse wave velocity; CFR = coronary flow reserve; ACPA = anticitrullinated peptide antibodies.

**Table 2:** ADMA lowering effect and possible pharmacodynamic mechanism of different drugs.

Drug	Investigated conditions	Hypothesized mechanism	Results	References
Statins	Diabetes mellitus, stroke, hypercholesterolemia	Increase DDAH expression, increased bioavailability of tetrahydrobiopterin	Decreased ADMA serum levels (18–50%)	[100, 109]
Fibrate	Hypertriglyceridemia	Increase DDAH activity through NF-kB suppression via PPAR-*α* receptors	Uncertain effect on ADMA serum levels, increase L-arginine/ADMA ratio	[111]
Niacine	Dyslipidemia	Depletion of methyl groups for niacine metabolism and consequent reduction in ADMA synthesis	Decreased ADMA serum levels (10%)	[112]
ACE inhibitors/ARB	Chronic glomerulonephritis, hypertension	Decreased NADPH oxidase upregulation by RAA system, with consequent reduced ROS-mediated DDAH inhibition	Decreased ADMA serum levels (10–16%)	[113, 114]
Thiazolidinediones	Diabetes mellitus	Through PPAR-*γ* receptor activation: reduced insulin resistance, increased expression of DDAH in renal tubules, suppressed activity of NF-kB	Controversial; from no reduction to reduction of ADMA serum levels (10%), possible protection against ADMA effect	[115]
Metformin	Diabetes mellitus Polycystic ovarian syndrome	Partially unknown, apparently not mediated by PRTM or DDAH Competitive antagonist of ADMA	Decreased ADMA serum levels (27%)	[116]
Nebivolol	Hypertension	Upregulation of DDAH, downregulation of PRTM	Decreased ADMA serum levels (37–44%)	[117, 118]
Acetylsalicylic acid	Coronary artery disease	Upregulation of DDAH and eNOS	Decreased ADMA serum levels (30%)	[119]
Estrogens	Postmenopausal women	Upregulation of DDAH via ER*α*	Decreased ADMA serum levels (18–20%)	[120, 121]
Folate and B group vitamins	Hypertension, hyperhomocysteinemia, chronic heart failure	Increased bioavailability of methylenetetrahydrofolate	Decreased ADMA serum levels (14%), acute decrease during e.v. infusion	[122, 123]
*α*-Lipoic acid	End-stage renal disease, diabetes mellitus	Activation and upregulation of DDAH via STAT3	Decreased ADMA serum levels (9%)	[124]
N-Acetylcysteine	End-stage renal disease	Partially unknown, direct activation DDAH, or ROS scavenging	Decreased ADMA serum levels (30%)	[125]
